# A new species of *Mollitrichosiphum* Suenaga from Taiwan Island (Hemiptera, Aphididae), based on morphological characteristics and DNA sequences

**DOI:** 10.3897/zookeys.524.6075

**Published:** 2015-09-30

**Authors:** Li-Yun Jiang, Jing Chen, Ge-Xia Qiao

**Affiliations:** 1 Key Laboratory of Zoological Systematics and Evolution, Institute of Zoology, Chinese Academy of Sciences, No. 1 Beichen West Road, Chaoyang District, Beijing 100101, P.R.China

**Keywords:** *Mollitrichosiphum tumorisiphum*, Greenideinae, morphology, DNA barcode, NJ tree

## Abstract

A new species of *Mollitrichosiphum* Suenaga, *Mollitrichosiphum tumorisiphum* Qiao & Jiang, **sp. n.**, from *Fagus longipetiolata* in Taiwan island is described. Siphunculi of *Mollitrichosiphum tumorisiphum* in alatae are distinctly swollen on the distal part, unlike those of the other known species in the genus. Updated keys to apterous and alate viviparous females of all known Chinese species of *Mollitrichosiphum* are provided. The specimens studied are deposited in the National Zoological Museum of China, Institute of Zoology, Chinese Academy of Sciences, Beijing, China and the Natural History Museum, London, United Kingdom.

## Introduction

The oriental genus *Mollitrichosiphum* Suenaga (Greenideinae, Aphididae) is restricted mainly to south-east Asia, and is represented by 11 known species in China (Remaudière and Remaudière 1997, Zhang and Qiao 2010). It is distinguishable from other genera of Greenideinae by a series of transverse ridges on the hind tibia. At present, there are six species recorded in Taiwan Island (Tao 1990, 1999, Zhang and Qiao 2010). Amongst aphid samples in the recent survey of Taiwan, two samples of the genus *Mollitrichosiphum* were found that could not be identified to any known species. Based on morphological features and molecular data, one new species *Mollitrichosiphum tumorisiphum* Qiao & Jiang, sp. n., feeding on *Fagus longipetiolata*, from mountainous areas in the northern and central part of Taiwan is described here. This new species differs from any other *Mollitrichosiphum* species in having alatae with siphunculi distinctly swollen on the distal part. Updated keys to the Chinese species of this genus are provided.

## Materials and methods

*Morphological description.* Aphid terminology and the measurements in this paper generally follow Blackman and Eastop (2006) and Zhang and Qiao (2010). The unit of measurement in this paper is millimetres (mm). Metrical data are listed in Table 1. The holotype and one alate viviparous female of the paratypes (No. 26510) are illustrated in Figures 1–36.

**Figures 1–12.**
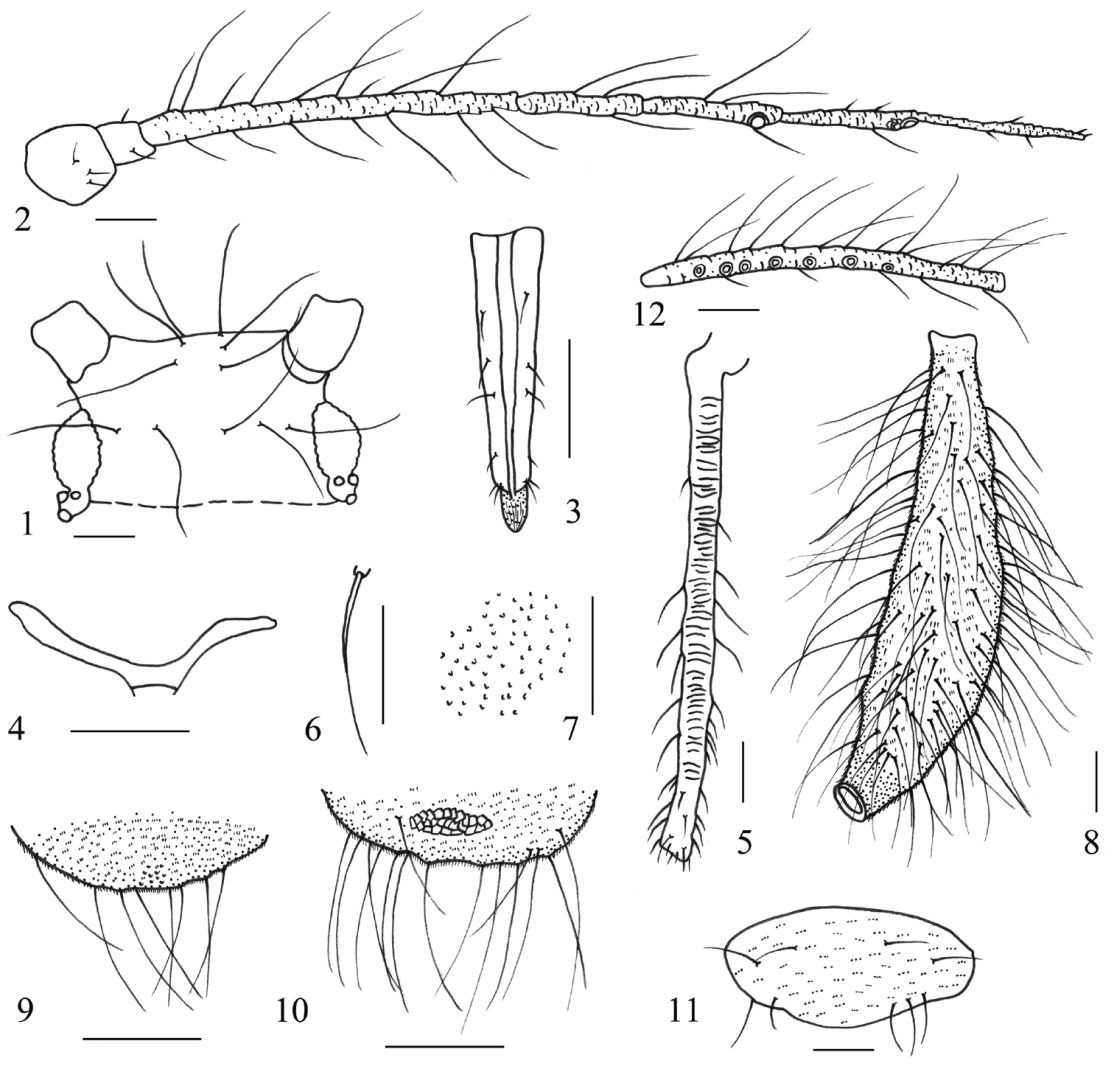
*Mollitrichosiphum tumorisiphum* Qiao & Jiang, sp. n. Apterous viviparous female: **1** dorsal view of head **2** antenna **3** ultimate rostral segment **4** mesosternal furca **5** hind tibia **6** dorsal seta on abdominal tergite I **7** spinules on venter of abdominal segment V **8** siphunculus **9** cauda **10** anal plate **11** genital plate. Alate viviparous female: 12. antennal segment III. Scale bars = 0.10 mm.

**Figures 13–26.**
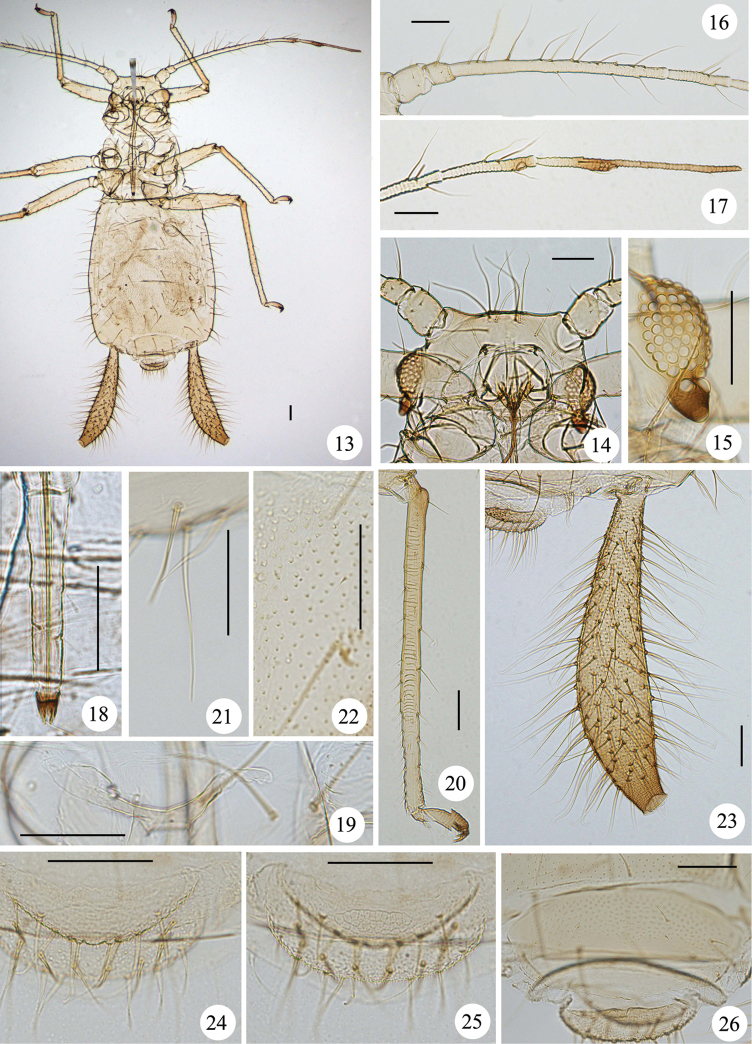
*Mollitrichosiphum tumorisiphum* Qiao & Jiang, sp. n. Apterous viviparous female: **13** dorsal view of body **14** dorsal view of head **15** compound eyes **16** antennal segments I–IV **17** antennal segments V–VI **18** ultimate rostral segment **19** mesosternal furca **20** hind tibia, tarsi and claws **21** dorsal seta on abdominal tergite I **22** spinules on venter of abdominal segment V **23** siphunculus **24** cauda; **25** anal plate **26** genital plate. Scale bars = 0.10 mm.

**Figures 27–37.**
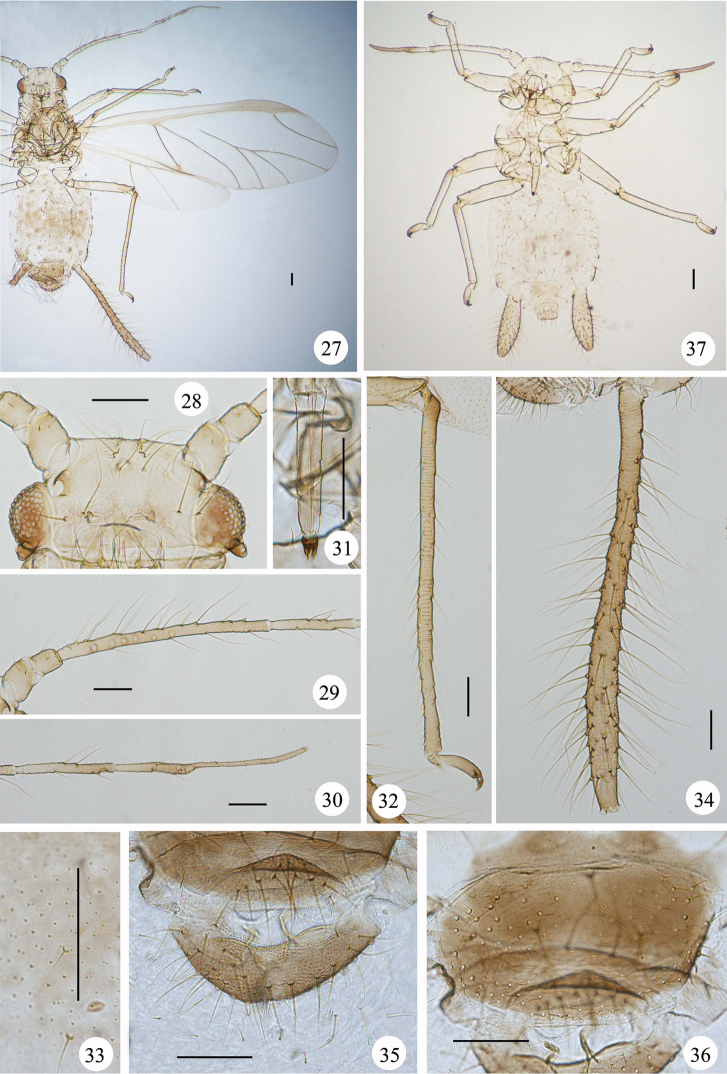
*Mollitrichosiphum tumorisiphum* Qiao & Jiang, sp. n. Alate viviparous female: **27** dorsal view of body **28** dorsal view of head **29** antennal segments I–IV **30** antennal segments V–VI **31** ultimate rostral segment **32** hind tibia, tarsi and claws **33** spinules on venter of abdominal segment V **34** siphunculus **35** cauda and anal plate **36** genital plate. Second instar larva: 37. dorsal view of body. Scale bars = 0.10 mm.

**Table 1.** Metrical data (mean, range and standard deviation) of *Mollitrichosiphum tumorisiphum* Qiao & Jiang, sp. n. (in mm, abbreviations see Materials and Methods). Ant. I, II, III, IV, V, VIb, antennal segments I, II, III, IV, V and the base of antennal segment VI, respectively; PT, processus terminalis; Ant. III BD, the basal diameter of antennal segment III; URS, ultimate rostral segment; URS BW, basal width of ultimate rostral segment; 2HT, second hind tarsal segment; Hind tibia MW, mid-width of hind tibia; SIPH, siphunculi; SIPH BW, basal width of siphunculi; SIPH DW, distal width of siphunculi; SIPH EW, width of expanded part on siphunculi; Cauda BW, basal width of cauda; Setae on Tergite I: marginal setae on abdominal tergite I; Setae on Tergite VIII: spinal setae on abdominal tergite VIII.

Parts		Apterous viviparae (n=5)			Alate viviparae (n=2)	
		Mean	Range	Standard deviation	Mean	Range
Length (mm)	Body length	2.14	1.92–2.34	0.12	2.11	2.07–2.15
	Body width	0.98	0.92–1.04	0.04	0.79	0.77–0.81
	Whole antennae	1.59	1.53–1.67	0.05	1.76	1.76
	Ant. I	0.11	0.11–0.12	0.002	0.11	0.11
	Ant. II	0.07	0.07	0	0.07	0.07
	Ant. III	0.54	0.51–0.59	0.03	0.62	0.58–0.67
	Ant. IV	0.18	0.16–0.21	0.01	0.24	0.23–0.25
	Ant. V	0.21	0.17–0.23	0.01	0.26	0.26
	Ant. VIb	0.20	0.19–0.21	0.005	0.21	0.21
	PT	0.27	0.26–0.29	0.01	0.30	0.30
	URS	0.21	0.18–0.22	0.01	0.21	0.21
	Hind femur	0.53	0.51–0.57	0.01	0.58	0.57–0.59
	Hind tibia	0.84	0.80–0.90	0.03	0.96	0.96
	2HT	0.13	0.125–0.134	0.004	0.13	0.13
	SIPH	0.77	0.73–0.86	0.03	1.16	1.12–1.2
	SIPH BW	0.07	0.07–0.09	0.007	0.07	0.06–0.09
	SIPH DW	0.07	0.06–0.08	0.005	0.05	0.048–0.053
	SIPH EW	0.16	0.13–0.19	0.02	0.10	0.09–0.12
	Cauda	0.05	0.05–0.07	0.008	0.05	0.05
	Cauda BW	0.19	0.18–0.20	0.006	0.18	0.17–0.18
	Ant. III BD	0.04	0.03–0.04	0.002	0.03	0.03
	Hind tibia MW	0.05	0.04–0.05	0.002	0.04	0.04
	Cephalic setae	0.18	0.16–0.19	0.01	0.16	0.15–0.16
	Setae on Tergite I	0.13	0.12–0.13	0.007	0.06	0.06–0.07
	Setae on Tergite VIII	0.09	0.08–0.12	0.01	0.13	0.12–0.13
	Setae on ANT. III	0.18	0.17–0.19	0.008	0.18	0.17–0.18
	Setae on Hind tibia	0.09	0.08–0.11	0.006	0.09	0.09–0.10
Ratio (times)	Whole antennae / Body	0.7	0.71–0.72	0.2	0.9	0.9
	Hind femur / Ant. III	1	0.9–1.0	0.03	0.9	0.9–1.0
	Hind tibia / Body	0.8	0.75–0.83	0.03	0.9	0.89–0.93
	PT / Ant. VIb	1.4	1.3–1.5	0.06	1.4	1.4
	URS / URS BW	5	4–6	0.6	5.5	5.5
	URS / 2HT	1.6	1.4–1.8	0.1	1.7	1.7
	Cauda / Cauda BW	0.3	0.2–0.4	0.04	0.3	0.26–0.28
	Cephalic setae / Ant. III BD	4.9	4.3–5.7	0.5	4.7	4.6–4.9
	Setae on Tergite I / Ant. III BD	3.5	3.3–4.0	0.2	1.9	1.7–2.0
	Setae on Tergite VIII / Ant. III BD	2.6	2.3–3.4	0.4	3.7	3.4–4.0
	Setae on ANT. III / ANT. III BD	5	4.5–5.7	0.4	5.3	5.1–5.4
	Setae on hind tibia / Hind tibia MW	2	1.8–2.2	0.1	2.4	2.2–2.5
	SIPH / Body	0.4	0.3–0.4	0.02	0.6	0.5–0.6
	SIPH / Ant. III	1.4	1.3–1.5	0.06	1.9	1.8–2.0
	SIPH / SIPH BW	10.7	8.7–12.6	1.12	16.7	13.9–19.5
	SIPH / SIPH DW	11.4	10. 7–13.0	0.8	23.1	21.3–25.0
	SIPH / SIPH EW	5.0	4.2–5.6	0.5	11.7	10.4–13.0

*Molecular analyses.* Fifty-seven samples belonging to eight *Mollitrichosiphum* species were included. The standard molecular barcode, mitochondrial cytochrome *c* oxidase subunit I (COI), and a faster-evolving gene, cytochrome *b*
(Cytb), were used. All sequences were taken from Liu et al. (2013), Zhang et al. (2011) and Zhang et al. (2012). Voucher information and GenBank accession numbers for all samples are listed in Table 2. Multiple alignments were conducted with ClustalX 2.0.12 (Larkin et al. 2007) and then verified manually. Neighbor-joining (NJ) trees and genetic distances were estimated for both COI and Cytb sequences with MEGA 6.06 (Tamura et al. 2013), using Kimura’s two-parameter (K2P) model (Kimura 1980). Bootstrap analyses were performed with 1000 replications.

*Specimen depositories*. The holotype, some paratypes of the new species and the other specimens examined are deposited in the National Zoological Museum of China, Institute of Zoology, Chinese Academy of Sciences, Beijing, China. Two paratypes (including to one apterous and one alate viviparous females) of the new species are deposited in the Natural History Museum (BMNH), London, the United Kingdom.

**Table 2.** Voucher information and GenBank accession numbers for aphid samples used in the molecular study.

Species	Voucher number	Host plant	Collection locality	COI	Cytb
*Mollitrichosiphum luchuanum* (Takahashi)	14414	*Amygdalus persica*	Fujian: Mt. Wuyi	JQ926108 P^a^	JF969358 P^b^
*Mollitrichosiphum luchuanum* (Takahashi)	14488	*Amygdalus persica*	Fujian: Mt. Wuyi	JQ926107 P^a^	JF969361 P^b^
*Mollitrichosiphum luchuanum* (Takahashi)	18104	*Meliosma rigida*	Fujian: Mt. Wuyi	JQ926105 P^a^	JF969368 P^b^
*Mollitrichosiphum luchuanum* (Takahashi)	21910	Unknown	Guangdong: Shixing	JQ926106 P^a^	JF969389 P^b^
*Mollitrichosiphum montanum* (van der Goot)	16504	Unknown	Tibet: Zhangmu	JQ926104 P^a^	JF969367 P^b^
*Mollitrichosiphum montanum* (van der Goot)	18324	Unknown	Tibet: Zayu	JQ926103 P^a^	JF969393 P^b^
*Mollitrichosiphum montanum* (van der Goot)	23754	*Alnus nepalensis*	Yunnan: Jingdong	JQ926102 P^a^	JF969387 P^b^
*Mollitrichosiphum nandii* Basu	14712	*Alnus cremastogyne*	Yunnan: Baoshan	JQ926101 P^a^	JF969364 P^b^
*Mollitrichosiphum nandii* Basu	15370	Unknown	Tibet: Medog	JQ926100 P^a^	JF969365 P^b^
*Mollitrichosiphum nandii* Basu	18382	*Fagus longipetiolata*	Tibet: Tangmai	JQ926099 P^a^	JF969369 P^b^
*Mollitrichosiphum nandii* Basu	23101	Unknown	Sichuan: Mt. Luoji	JQ926148 P^a^	JF969394 P^b^
*Mollitrichosiphum nigrofasciatum* (Maki)	14560	*Lithocarpus glaber*	Fujian: Mt. Wuyi	JQ926098 P^a^	JF969363 P^b^
*Mollitrichosiphum nigrofasciatum* (Maki)	14805	*Cyclobalanopsis glauca*	Fujian: Mt. Wuyi	JQ926097 P^a^	JF969395 P^b^
*Mollitrichosiphum nigrofasciatum* (Maki)	17329	*Quercus* sp.	Zhejiang: Taishun	JQ926096 P^a^	JN645006 P^c^
*Mollitrichosiphum nigrofasciatum* (Maki)	17331	Fagaceae	Zhejiang: Taishun	JQ926095 P^a^	NA
*Mollitrichosiphum nigrofasciatum* (Maki)	17333	Fagaceae	Zhejiang: Taishun	JQ926094 P^a^	NA
*Mollitrichosiphum nigrofasciatum* (Maki)	17387	*Quercus aliena*	Zhejiang: Taishun	JQ926093 P^a^	NA
*Mollitrichosiphum nigrofasciatum* (Maki)	18499	*Castanopsis* sp.	Hainan: Mt. Diaoluo	JQ926092 P^a^	NA
*Mollitrichosiphum nigrofasciatum* (Maki)	18510	*Lithocarpus elmerrillii*	Hainan: Mt. Diaoluo	JQ926090 P^a^	JN645010 P^c^
*Mollitrichosiphum nigrofasciatum* (Maki)	21773	Unknown	Hunan: Mt. Bamian	JQ926089 P^a^	NA
*Mollitrichosiphum nigrofasciatum* (Maki)	21859	Unknown	Guangdong: Ruyuan	JQ926088 P^a^	NA
*Mollitrichosiphum nigrofasciatum* (Maki)	21916	Elaeocarpaceae	Guangdong: Shixing	JQ926087 P^a^	NA
*Mollitrichosiphum nigrofasciatum* (Maki)	21966	Unknown	Guangdong: Shixing	JQ926086 P^a^	JF969399 P^b^
*Mollitrichosiphum nigrofasciatum* (Maki)	22101	*Lithocarpus glaber*	Fujian: Longyan	JQ926085 P^a^	JF969400 P^b^
*Mollitrichosiphum nigrum* Zhang & Qiao	14405	*Castanea* sp.	Fujian: Mt. Wuyi	JQ926083 P^a^	JN645004 P^c^
*Mollitrichosiphum nigrum* Zhang & Qiao	14417	*Elaeagnus pungens*	Fujian: Mt. Wuyi	JQ926084 P^a^	JF969359 P^b^
*Mollitrichosiphum nigrum* Zhang & Qiao	18913	*Meliosma cuneifolia*	Guangxi: Longsheng	JQ926082 P^a^	JF969375 P^b^
*Mollitrichosiphum nigrum* Zhang & Qiao	19258	*Ailanthus altissima*	Guangxi: Xing’an	JQ926081 P^a^	JF969377 P^b^
*Mollitrichosiphum nigrum* Zhang & Qiao	21845	Unknown	Hunan: Mt. Mang	JQ926080 P^a^	JF969390 P^b^
*Mollitrichosiphum nigrum* Zhang & Qiao	21856	Unknown	Guangdong: Ruyuan	JQ926079 P^a^	JF969391 P^b^
*Mollitrichosiphum nigrum* Zhang & Qiao	21872	Unknown	Guangdong: Ruyuan	JQ926078 P^a^	JN645011 P^c^
*Mollitrichosiphum rhusae* Ghosh	18508	*Helicia hainanensis*	Hainan: Mt. Diaoluo	JQ926077 P^a^	JF969371 P^b^
*Mollitrichosiphum rhusae* Ghosh	18511	*Helicia hainanensis*	Hainan: Mt. Diaoluo	JQ926076 P^a^	JF969372 P^b^
*Mollitrichosiphum rhusae* Ghosh	18513	Fagaceae	Hainan: Mt. Diaoluo	JQ926075 P^a^	JF969373 P^b^
*Mollitrichosiphum rhusae* Ghosh	18514	*Helicia hainanensis*	Hainan: Mt. Diaoluo	JQ926074 P^a^	JF969374 P^b^
*Mollitrichosiphum rhusae* Ghosh	20811	Fagaceae	Hainan: Mt. Wuzhi	JQ926073 P^a^	JF969380 P^b^
*Mollitrichosiphum rhusae* Ghosh	20858	Meliaceae	Hainan: Mt. Diaoluo	JQ926072 P^a^	JF969381 P^b^
*Mollitrichosiphum tenuicorpus* (Okajima)	14421	*Castanea* sp.	Fujian: Mt. Wuyi	JQ926070 P^a^	JF969360 P^b^
*Mollitrichosiphum tenuicorpus* (Okajima)	14537	*Castanopsis sclerophylla*	Fujian: Mt. Wuyi	JQ926069 P^a^	JF969362 P^b^
*Mollitrichosiphum tenuicorpus* (Okajima)	18506	*Cyclobalanopsis neglecta*	Hainan: Mt. Diaoluo	JQ926067 P^a^	JF969370 P^b^
*Mollitrichosiphum tenuicorpus* (Okajima)	18614	*Castanopsis carlesii*	Guangdong: Shixing	JQ926066 P^a^	JF969396 P^b^
*Mollitrichosiphum tenuicorpus* (Okajima)	18892	Fagaceae	Guangxi: Longsheng	JQ926065 P^a^	JF969397 P^b^
*Mollitrichosiphum tenuicorpus* (Okajima)	19242	Fagaceae	Hainan: Mt. Bawang	JQ926064 P^a^	JF969376 P^b^
*Mollitrichosiphum tenuicorpus* (Okajima)	19521	*Quercus* sp.	Hainan: Mt. Jianfeng	JQ926063 P^a^	JF969378 P^b^
*Mollitrichosiphum tenuicorpus* (Okajima)	20530	*Castanopsis ferox*	Yunnan: Simao	JQ926062 P^a^	JF969379 P^b^
*Mollitrichosiphum tenuicorpus* (Okajima)	20866	Fagaceae	Hainan: Mt. Jianfeng	JQ926061 P^a^	JF969382 P^b^
*Mollitrichosiphum tenuicorpus* (Okajima)	20938	*Castanopsis fabri*	Hainan: Mt. Jianfeng	JQ926060 P^a^	JF969383 P^b^
*Mollitrichosiphum tenuicorpus* (Okajima)	22152	Unknown	Fujian: Zhangzhou	JQ926059 P^a^	JF969384 P^b^
*Mollitrichosiphum tenuicorpus* (Okajima)	22155	Unknown	Fujian: Zhangzhou	JQ926058 P^a^	JF969385 P^b^
*Mollitrichosiphum tenuicorpus* (Okajima)	22161	Unknown	Fujian: Zhangzhou	JQ926057 P^a^	JN645013 P^c^
*Mollitrichosiphum tenuicorpus* (Okajima)	22166	Unknown	Fujian: Zhangzhou	JQ926056 P^a^	JF969386 P^b^
*Mollitrichosiphum tenuicorpus* (Okajima)	23843	*Castanopsis hystrix*	Yunnan: Cangyuan	JQ926055 P^a^	JX186736 P^a^
*Mollitrichosiphum tenuicorpus* (Okajima)	26029	*Castanopsis eyrei*	Guangxi: Lingui	JN644999 P^c^	JN645015 P^c^
*Mollitrichosiphum tenuicorpus* (Okajima)	26261	*Castanopsis* sp.	Guangxi: Mt. Shiwandashan	JN645000 P^c^	JN645016 P^c^
*Mollitrichosiphum tenuicorpus* (Okajima)	26270	*Castanopsis* sp.	Guangxi: Mt. Shiwandashan	JQ418313 P^c^	JQ418317 P^c^
*Mollitrichosiphum tumorisiphum* Qiao & Jiang, sp. n.	26510	*Fagus longipetiolata*	Taiwan: Mt. Taman	JN645002 P^c^	JQ418315 P^c^
*Mollitrichosiphum tumorisiphum* Qiao & Jiang, sp. n.	26515	*Fagus longipetiolata*	Taiwan: Hualian	JN645003 P^c^	JQ418316 P^c^

Reference sequences form previous studies: P^a^P Liu et al. (2013), P^b^P Zhang et al. (2011), P^c^P Zhang et al. (2012).

## Taxonomy

### Mollitrichosiphum (Metatrichosiphum) tumorisiphum

Taxon classificationAnimaliaHemipteraAphididae

Qiao & Jiang sp. n.

http://zoobank.org/D85E577E-D2FA-40CA-84AD-112101E86024

#### Descriptions.

**Figures 38–41.**
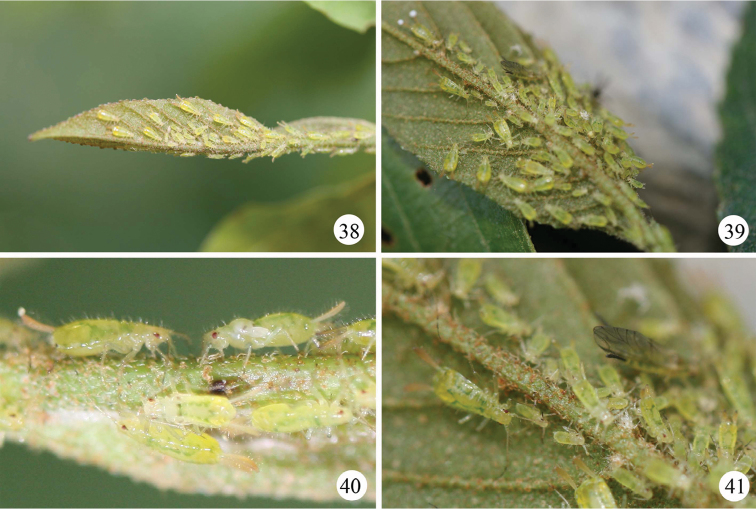
*Mollitrichosiphum tumorisiphum* Qiao & Jiang, sp. n. **38** colony on the bud of the host **39** colony on the underside of the leaf **40** apterous viviparous female and larvae **41** apterous, alate viviparous females and larvae.

*Apterous viviparous female*: Body elongated oval (Fig. 13), yellow green in life, with pairs of emerald green dorsal markings and pale brown siphunculi (Figs 38–41).

**Mounted specimens.** Body pale brown, with head and prothorax fused. Dorsal setae thick, long and pointed (Figs 6, 21).

**Head.** Ocular tubercles dark brown, well developed. Dorsum of head with three pairs of setae between antennae, and 4–6 setae between eyes. Maximum lengths of cephalic setae 4.3–5.7 times as long as basal diameter of antennal segment III. Front flat, antennal tubercles slightly developed (Figs 1, 14). Antennae 6-segmented (Figs 2, 16–17), 0.71-0.72 times as long as body length. Processus terminalis 1.3–1.5 times as long as base of the segment. Antennal segments I–IV, basal half of segment V and of base of segment VI pale brown, other parts of segment V and VI brown. Antennal segments III–VI with short imbrications. Antennal setae pointed. Antennal segment I with only short setae; segments II–V with long and short setae, setae on the inner side of the segment distinctly longer, thicker and more numerous than setae on the outer side of the segment; segments I–VI each with 4–6, 4 or 5, 16–23, 3 or 4, 3 or 4, (3 or 4)+(4–6) setae, respectively; apex of processus terminalis with 3 or 4 short blunt setae; maximum length of setae on segment III 4.5–5.7 times as long as basal diameter of the segment. Rostrum reaching hind coxae, sometimes abdominal segment I; ultimate rostral segment pale brown, except for brown apex, long and wedge-shaped (Figs 3, 18), 4–6 times as long as its basal width, 1.4–1.8 times as long as second hind tarsal segment; segment IV and V obviously separated; with 3 pairs of primary and 3 pairs of secondary setae.

**Thorax.** Mesosternal furca with a short stem (Figs 4, 19). Pronotum with 1 pair of anterior spinal, 3 or 4 posterior spinal, 1 pair of anterior marginal and 2 pairs of posterior marginal setae Legs slender. Femora and tibiae pale brown. Hind femur 0.9–1 times as long as antennal segment III. Hind tibia 0.75–0.83 times as long as body, with 57–62 transverse ridges on basal 3/4 of the segment (Figs 5, 20). Setae on legs short, pointed or acuminate. Maximum length of setae on hind tibia 1.8–2.2 times as long as mid-width of the segment. Tarsi brown, with transverse imbrications. Chaetotaxy of first tarsomeres: 7, 7, 7.

**Abdomen.** Abdominal tergite I with 4–6 spinal and pleural setae, and 2 pairs of marginal setae, tergite VIII with one pair of spinal setae. Maximum lengths of marginal setae on abdominal tergite I and dorsal setae on tergite VIII 3.3–4.0 and 2.3–3.4 times as long as basal diameter of antennal segment III, respectively. Venter of abdominal segments II–VI with coarse spinules on pleural and sub-marginal area (Figs 7, 22). Spiracles oval and open, on pale brown spiracular plates. Siphunculi brown, long and tubular, strongly swollen over most of length and constricted near apex (Figs 8, 23), flange distinct; 0.3–0.4 times as long as body, 1.3–1.5 times as long as antennal segment III, 8.7–12.6 times as long as its basal width, 4.2–5.6 times as long as width of expanded part, 10.7–13 times as long as its distal width. Siphunculi with spinules evenly distributed and with spinulose imbrications at apex. Each siphunculus with 95–118 setae, long and pointed. Cauda, anal plate and genital plate pale brown. Cauda broadly rounded (Figs 9, 24), with spinules and round apex; 0.2–0.4 times as long as its basal width; with 8–10 setae. Anal plate transversely elliptical (Figs 10, 25), with spinules and with a transverse band of cell-like markings on spinal area, with 16 or 17 setae. Genital plate transverse oval (Figs 11, 26), with spinules, 4–6 anterior and 4–8 posterior setae. Gonapophyses three, spinal one with 6 setae and each pleural one with 3 setae.

*Alate viviparous female*: Body elongate oval (Fig. 27), yellow green in life, with green dorsal markings (Figs 39, 41), dark brown forewing veins and dark brown siphunculi (Fig. 41).

**Mounted specimens.** Dorsal setae thick, long and pointed.

**Head.** Head, antennae and ultimate rostral segment dark brown. Dorsum of head with 6 setae between antennae, and 4–6 setae between eyes. Maximum lengths of cephalic setae 4.6–4.9 times as long as basal diameter of antennal segment III. Front flat (Fig. 28). Antennae 6-segmented (Figs 12, 29–30), 0.9 times as long as body length. Processus terminalis 1.4 times as long as base of the segment. Antennal segments III–VI with short imbrications. Antennal setae thick, long and pointed; segments I–VI each with 4, 4 or 5, 18, 4 or 5, 4, 4+5 setae, respectively; apex of processus terminalis with 4 short blunt setae; length of setae on segment III 5.1–5.4 times as long as basal diameter of the segment. Antennal segment III with 7–9 nearly round secondary rhinaria, distributed on basal 2/3 of the segment. Rostrum reaching abdominal segment I; ultimate rostral segment long wedge-shaped (Fig. 31), 5.5 times as long as its basal width, 1.7 times as long as second hind tarsal segment; segment IV and V obviously separated; with 3 pairs of primary and 2-3 pairs of secondary setae.

**Thorax.** Thorax, femora, tibiae and tasi dark brown. Pronotum with 6 spinal and pleural setae and 3 pairs of marginal setae. Legs slender. Inside of distal half of femora with short spare spinulose imbrications. Hind femur 0.9–1 times as long as antennal segment III. Hind tibia 2 times as long as body, with 69–72 transverse ridges on basal 3/4 of the segment (Fig. 33). Setae on legs short and pointed. Maximum length of setae on hind tibia 2.5–2.9 times as long as mid-width of the segment. Second tarsal segments with transverse imbrications. Chaetotaxy of first tarsomeres: 7, 7, 7. Fore wings with media twice branched and distal 1/3 of CuR_1 _Rcurved to media; hind wings with 2 oblique veins.

**Abdomen.** Abdominal tergites I–VI with spinal, pleural and marginal sclerotic markings fused into a large brown patch; tergites VII and VIII each with one brown transverse patch. Abdominal tergite I with 8–10 setae, tergite VII with 4 setae, tergite VIII with 2 setae. Maximum lengths of marginal setae on abdominal tergite I and dorsal setae on tergite VIII 1.7–2.0 and 3.4–4.0 times as long as basal diameter of antennal segment III, respectively. Venter of abdominal segments III–VI with coarse spinules on pleural and sub-marginal area. Spiracles oval and open, on brown oval spiracular plates. Siphunculi long. tubular, distinctly swollen on distal half (Fig. 34), flange distinct, basal 2/3 of siphunculi dark brown and distal 1/3 brown, with spinules evenly distributed and with spinulose imbrications at apex; 0.5–0.6 times as long as body, 1.8–2 times as long as antennal segment III, 13.9–19.5 times as long as its basal width, 10.4–13 times as long as width of expanded part, 21.3–25 times as long as its distal width; each with 105–120 long and pointed setae. Cauda, anal plate and genital plate brown. Cauda broadly rounded (Fig. 35); 0.26–0.28 times as long as its basal width; with spare spinulose imbrications and 12 setae. Anal plate transversely elliptical, with spare spinulose imbrications and 52–58 setae (Fig. 35). Genital plate transverse oval (Fig. 36), with spinules and 84–104 setae. Gonapophyses three, spinal one with 8 setae and each pleural one with 4 setae.

#### Specimens examined.

Holotype: apterous viviparous female, **CHINA**, Taiwan Island: Tamanshan Mountain, Fuxing Town, Taoyuan County, 24.70°N, 121.43°E, altitude 1630m, 14 June 2011, No. 26510–1–1–1, on *Fagus longipetiolata*, coll. X.L. Huang. Paratypes: 3 apterous viviparous females, 1 alate viviparous females and 1 second instar larva, 1 apterous viviparous female and 1 alate viviparous female (BMNH), with the same collection data as holotype; 1 apterous viviparous female, Bilu, Xiulin Town, Hualian County, 24.00°N, 121.21°E, altitude 2150m, 20 July 2011, No. 26515, on *Fagus longipetiolata*, coll. X.L. Huang.

#### Etymology.

The name of this species is derived from its most distinctive feature, its markedly swollen siphunculi. The specific name is composed of “*tumor* (Latin, =inflated, swelling)” and “*siphum* (Latin, =tube)”.

#### Diagnosis.

The new species is distinctly different from the other known species in the genus, based on siphunculi of alatae distinctly expanded on the apical half. It is similar to the species Mollitrichosiphum (Metatrichosiphum) niitakaensis (Takahashi), but differs from the latter as follows: body with long and stout dorsal setae, pointed at apex (the latter: at least with some bifurcate dorsal setae); each siphunculus with more than 95 setae (the latter: less than 80); hind tibia with 20–63 short transverse ridges (the latter: with more than 84).

It is also similar to the species Mollitrichosiphum (Metatrichosiphum) yamabiwae Suenaga, but differs from the latter as follows in apterous viviparae: dorsal of abdomen pale brown, without sclerotic pattern (the latter: with fused dark brown sclerotic patterns); hind tibia with 57–62 transverse ridges (the latter: with 31–33); ultimate rostral segment 0.18–0.22 mm long, 4–6 times as long as basal width, 1.4–1.8 times as long as hind second tarsal segment (the latter: 0.28–0.41 mm, 6.1–8.4 times, 2.4–2.8 times); ultimate rostral segment with 3 pairs of secondary setae (the latter: with 7 pairs).

#### Biology.

Colonizing the underside of young leaves of new growth of *Fagus longipetiolata*. (Figs 38–41).

## Molecular analyses

**Figure 42.**
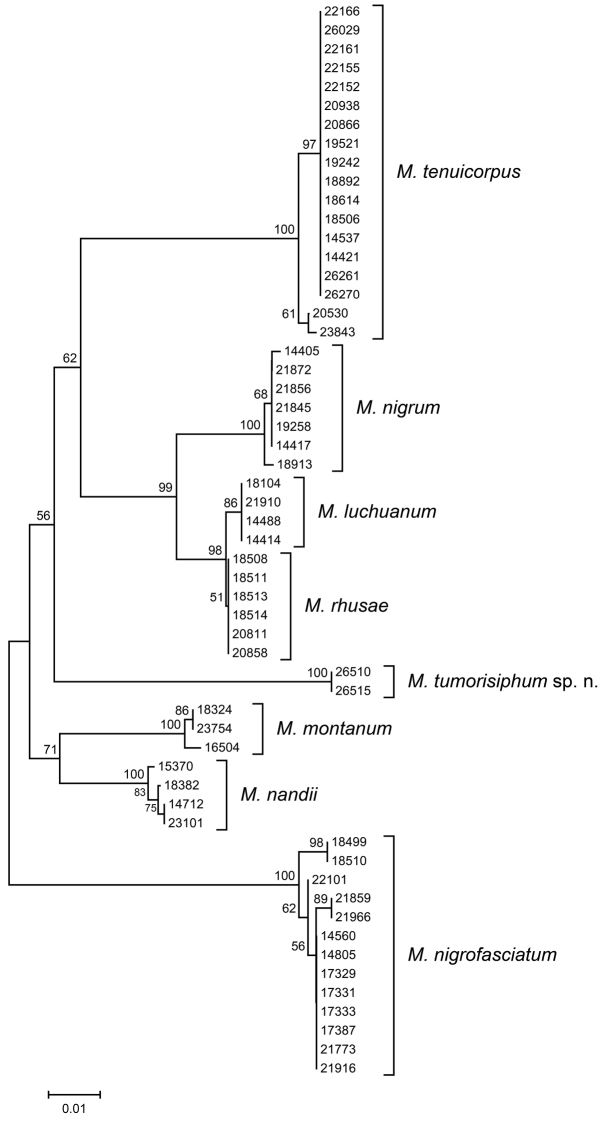
Neighbour-joining tree for *Mollitrichosiphum* samples based on COI sequences. Numbers above branches indicate bootstrap values (>50%).

**Figure 43.**
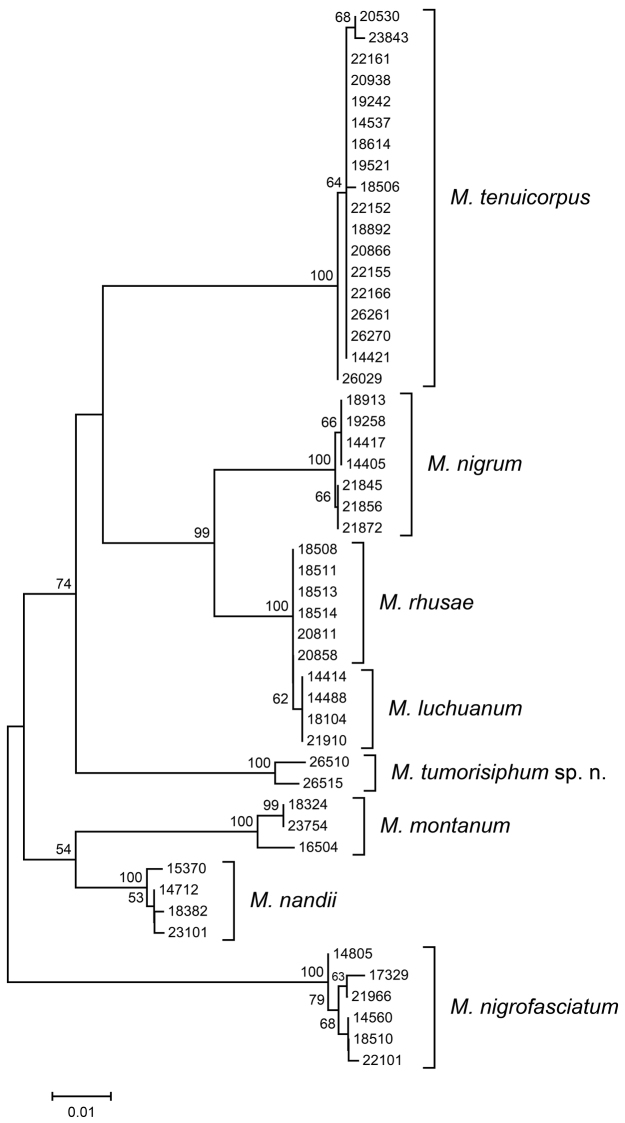
Neighbour-joining tree for *Mollitrichosiphum* samples based on Cytb sequences. Numbers above branches indicate bootstrap values (>50%).

The alignment sequences of COI and Cytb genes included 658 and 666 sites, of which 133 and 129 were parsimony-informative, respectively. The results of NJ analyses of COI and Cytb sequences are summarized. The NJ trees presented here are unrooted and do not reflect phylogenetic relationships, but are used to represent the genetic distance matrices (Foottit et al. 2008). The COI tree (Fig. 42) contained 57 samples of *Mollitrichosiphum* species and showed eight well-supported clades. All morphologically identified species, including *Mollitrichosiphum tumorisiphum* Qiao & Jiang, sp. n., formed monophyletic clusters, indicating that they are genetically distinct from one another. The Cytb tree (Fig. 43) containing 50 samples yielded a similar result, with *Mollitrichosiphum tumorisiphum* Qiao & Jiang, sp. n. again retrieved in a distinct clade.

For the sampled known species of *Mollitrichosiphum*, the mean intraspecific variation was 0.2% (range: 0–0.9%) in COI and 0.1% (range: 0–1.1%) in Cytb. The genetic distance between two distinct samples of *Mollitrichosiphum tumorisiphum* Qiao & Jiang, sp. n. was 0 in COI and 0.9% in Cytb. Interspecific genetic divergence between the known *Mollitrichosiphum* species averaged 8.7% (range: 0.3–12.5%) in COI and 8.1% (range: 0.2–12.3%) in Cytb. Pairwise sequence divergences of COI and Cytb among the *Mollitrichosiphum* species are presented in Table 3. The mean genetic distance between *Mollitrichosiphum tumorisiphum* Qiao & Jiang, sp. n. and the other *Mollitrichosiphum* species was 10.3% (range: 8.9–11.9%) in COI and 8.8% (range: 7.6%–11.0%) in Cytb, corresponding well to the interspecific divergence between the other known *Mollitrichosiphum* species.

**Table 3.** Kimura’s two-parameter genetic distances (mean ± standard deviation) among *Mollitrichosiphum* species based on COI (lower half of matrix) and Cytb (upper half of matrix) sequences. The genetic distances between *Mollitrichosiphum tumorisiphum* Qiao & Jiang, sp. n. and the other *Mollitrichosiphum* species are shown in bold.

Species	1	2	3	4	5	6	7	8
1. *Mollitrichosiphum luchuanum*		0.075±0.001	0.060±0.002	0.104±0.002	0.036±0.001	0.002±0	0.076±0.001	**0.082±0.001**
2. *Mollitrichosiphum montanum*	0.074±0.001		0.050±0.003	0.094±0.002	0.081±0.003	0.077±0.001	0.104±0.001	**0.089±0.001**
3. *Mollitrichosiphum nandii*	0.066±0.001	0.045±0.003		0.097±0.002	0.068±0.001	0.058±0.002	0.077±0.001	**0.078±0.002**
4. *Mollitrichosiphum nigrofasciatum*	0.102±0.001	0.089±0.002	0.095±0.002		0.114±0.002	0.102±0.002	0.117±0.002	**0.106±0.002**
5. *Mollitrichosiphum nigrum*	0.030±0.001	0.074±0.001	0.065±0.001	0.103±0.002		0.035±0.001	0.082±0.001	**0.094±0.001**
6. *Mollitrichosiphum rhusae*	0.003±0	0.074±0.001	0.063±0.001	0.100±0.001	0.030±0.001		0.075±0.001	**0.080±0.001**
7. *Mollitrichosiphum tenuicorpus*	0.077±0	0.088±0.001	0.082±0.002	0.121±0.003	0.084±0.001	0.074±0		**0.085±0.002**
8. *Mollitrichosiphum tumorisiphum* Qiao & Jiang, sp. n.	**0.096±0**	**0.090±0.001**	**0.094±0.001**	**0.117±0.002**	**0.095±0.001**	**0.096±0**	**0.105±0**	

The results of NJ analyses and genetic distances based on COI and Cytb sequences strongly confirmed that the new morphologically determined species *Mollitrichosiphum tumorisiphum* Qiao & Jiang, sp. n. was genetically different from the known *Mollitrichosiphum* species sampled in this study.

### Updated key to species of *Mollitrichosiphum* from China

**(Apterous viviparous females)**

**Table:** 

1	Antennal setae with similar length on inner and outer sides of the segment; hind tibia with 17–22 transverse ridges	**Mollitrichosiphum (Mollitrichosiphum) tenuicorpus (Okajima)**
–	Antennal setae long or short, long setae being mainly on the inner side of the segment; hind tibia with 20–84 transverse ridges	**2 Mollitrichosiphum (Metatrichosiphon) spp.**
2	Hind tibia with more than 84 short transverse ridges	**Mollitrichosiphum (Metatrichosiphum) niitakaensis (Takahashi)**
–	Hind tibia with 20–63 short transverse ridges	**3**
3	Abdominal tergite VII with 13 or 14 setae; body with pointed and dense dorsal setae	**Mollitrichosiphum (Metatrichosiphum) nandii Basu**
–	Abdominal tergite VII with only 2–4 setae	**4**
4	Siphunculi long, 0.7–0.9 times as long as body	**Mollitrichosiphum (Metatrichosiphum) montanum (van der Goot)**
–	Siphunculi at most 0.7 times as long as body	**5**
5	Body with long and stout dorsal setae, pointed at apex	**6**
–	Body at least with some bifurcate dorsal setae	**7**
6	Dorsal of abdomen with fused dark brown sclerotic patterns; hind tibia with 31–33 transeverse ridges; ultimate rostral segment 0.3–0.4 mm long, 6.1–8.4 times as long as basal width, 2.4–2.8 times as long as hind second tarsal segment, with 7 pairs of secondary setae	**Mollitrichosiphum (Metatrichosiphum) yamabiwae Suenaga**
–	Dorsal of abdomen pale brown, without sclerotic pattern; hind tibia with 57–62 transeverse ridges; ultimate rostral segment 0.18–0.22 mm long, 4–6 times as long as basal width, 1.36–1.77 times as long as hind second tarsal segment, with 3 pairs of secondary setae	**Mollitrichosiphum (Metatrichosiphum) tumorisiphum Qiao & Jiang, sp. n.**
7	Length of ultimate rostral segment less than 2 times that of hind second tarsal segment length	**8**
–	Length of ultimate rostral segment more than 2 times that of hind second tarsal segment length	**9**
8	Body 2.9 mm long; hind tibia with 37–43 transverse ridges	**Mollitrichosiphum (Metatrichosiphum) glaucae Takahashi**
–	Body 1.4–2.2 mm long; hind tibia with 27–38 transverse ridges	**Mollitrichosiphum (Metatrichosiphum) nigrofasciatum (Maki)**
9	Hind tibia with less than 30 transverse ridges	**10**
–	Hind tibia with more than 30 transverse ridges	**11**
10	Body setae mostly pointed; ultimate rostral segment 2.3–2.4 times as long as hind second tarsal segment; on plants of Fagaceae	**Mollitrichosiphum (Metatrichosiphum) luchuanum (Takahashi)**
–	Body setae mostly bifurcate; ultimate rostral segment 1.8	**Mollitrichosiphum (Metatrichosiphum) taiwanum (Takahashi)**
11	Body pale in mounted specimens, except for brown siphunculi; processus terminalis 1.6–1.8 times as long as the base of antennal segment VI; hind tibia with 35–46 transverse ridgest	**Mollitrichosiphum (Metatrichosiphum) rhusae Ghosh**
–	Body brown in mounted specimens; processus terminalis 1.3–1.6 times as long as the base of antennal segment VI; hind tibia with 53–63 transverse ridges	**Mollitrichosiphum (Metatrichosiphum) nigrum Zhang & Qiao**

**(Alate viviparous females)**

(Remark: *Mollitrichosiphum glaucae* and *Mollitrichosiphum niitakaensis* are not included in the key to alatae, because no specimens are available).

**Table:** 

1	Antennal setae on flagellum with similar length on inner and outer sides of the segment	**Mollitrichosiphum (Mollitrichosiphum) tenuicorpus (Okajima)**
–	Antennal setae on flagellum long or short, long setae mainly on the inner side of the segment	**2 Mollitrichosiphum (Metatrichosiphon) spp.**
2	Abdominal tergite VII with 9–12 setae	**Mollitrichosiphum (Metatrichosiphum) nandii Basu**
–	Abdominal tergite VII with 2–6 setae	**3**
3	Antennal segment III with 5–10 secondary rhinaria	**4**
–	Antennal segment III with more than 14 secondary rhinaria	**5**
4	Hind tibia with 25–43 transverse ridges; ultimate rostral segment 3.9–4.5 times as long as its basal width; each siphunculus with 65–96 setae	**Mollitrichosiphum (Metatrichosiphum) nigrofasciatum (Maki)**
–	Hind tibia with 69–72 transverse ridges; ultimate rostral segment 5.5 times as long as its basal width; each siphunculus with 105–120 setae	**Mollitrichosiphum (Metatrichosiphum) tumorisiphum Qiao & Jiang, sp. n.**
5	Hind tibia with more than 42 transverse ridges	**6**
–	Hind tibia with less than 38 transverse ridges	**8**
6	Ultimate rostral segment 1.8–1.9 times as long as hind second tarsal segment	**Mollitrichosiphum (Metatrichosiphum) montanum (van der Goot)**
–	Ultimate rostral segment more than 2.4 times of hind second tarsal segment length	**7**
7	Hind tibia with 49–53 transverse ridges; antennal segment III with 14–16 secondary rhinaria	**Mollitrichosiphum (Metatrichosiphum) nigrum Zhang & Qiao**
–	Hind tibia with about 43 transverse ridges; antennal segment III with 20 secondary rhinaria	**Mollitrichosiphum (Metatrichosiphum) rhusae Ghosh**
8	Hind tibia with about 30 transverse ridges; antennal segment III with 20 or 21 secondary rhinaria; siphunculi 0.8 times as long as body length	**Mollitrichosiphum (Metatrichosiphum) taiwanum (Takahashi)**
–	Hind tibia with about 34 transverse ridges; antennal segment III with less than 20 secondary rhinaria; siphunculi at most 0.8 times as long as body length	**9**
9	Ultimate rostral segmentIV about 5.2 times as long as segment V; siphunculi about 2.4 mm long, about 17.3 times as long as its basal width	**Mollitrichosiphum (Metatrichosiphum) luchuanum (Takahashi)**
–	Ultimate rostral segment IV 6.4–-7.0 times as long as segment V; siphunculi 1.7–1.8 mm long, 14.6–17.0 times as long as its basal width	**Mollitrichosiphum (Metatrichosiphum) yamabiwae Suenaga**

## Supplementary Material

XML Treatment for Mollitrichosiphum (Metatrichosiphum) tumorisiphum
